# The Effect of Substituted Benzene-Sulfonamides and Clinically Licensed Drugs on the Catalytic Activity of CynT2, a Carbonic Anhydrase Crucial for *Escherichia coli* Life Cycle

**DOI:** 10.3390/ijms21114175

**Published:** 2020-06-11

**Authors:** Sonia Del Prete, Viviana De Luca, Silvia Bua, Alessio Nocentini, Vincenzo Carginale, Claudiu T. Supuran, Clemente Capasso

**Affiliations:** 1 Institute of Biosciences and Bioresources, CNR, Via Pietro Castellino 111, 80131 Napoli, Italy; sonia.delprete@ibbr.cnr.it (S.D.P.); vivianadeluca.81@gmail.com (V.D.L.); vincenzo.carginale@cnr.it (V.C.); 2 Proteomics & Mass Spectrometry Laboratory, ISPAAM, CNR, Via Argine 1085, 80147 Naples, Italy; 3 Section of Pharmaceutical and Nutraceutical Sciences, Department of Neurofarba, University of Florence, Via U. Schiff 6, Sesto Fiorentino, 50019 Florence, Italy; silvia.bua@unifi.it (S.B.); alessio.nocentini@unifi.it (A.N.)

**Keywords:** carbonic anhydrase, sulfonamides, inhibitors, antibacterials, *Escherichia coli*, stopped-flow assay, protonography

## Abstract

Proteins are relevant antimicrobial drug targets, and among them, enzymes represent a significant group, since most of them catalyze reactions essential for supporting the central metabolism, or are necessary for the pathogen vitality. Genomic exploration of pathogenic and non-pathogenic microorganisms has revealed genes encoding for a superfamily of metalloenzymes, known as carbonic anhydrases (CAs, EC 4.2.1.1). CAs catalyze the physiologically crucial reversible reaction of the carbon dioxide hydration to bicarbonate and protons. Herein, we investigated the sulfonamide inhibition profile of the recombinant *β*-CA (CynT2) identified in the genome of the Gram-negative bacterium *Escherichia coli*. This biocatalyst is indispensable for the growth of the microbe at atmospheric pCO_2_. Surprisingly, this enzyme has not been investigated for its inhibition with any class of CA inhibitors. Here, we show that CynT2 was strongly inhibited by some substituted benzene-sulfonamides and the clinically used inhibitor sulpiride (K_I_s in the range of 82–97 nM). This study may be relevant for identifying novel CA inhibitors, as well as for another essential part of the drug discovery pipeline, such as the structure–activity relationship for this class of enzyme inhibitors.

## 1. Introduction

The first wholly sequenced microbial genomes were obtained in 1995 from two pathogenic bacteria, *Haemophilus influenzae* and *Mycoplasma genitalium* [1,2]. From 1995 onward, genomes belonging to 11,691 eukaryotes, 247,392 prokaryotes, and 34,747 viruses have been sequenced (Data from National Center for Biotechnology Information, May 2020). The extensive DNA sequencing has opened a new era to contrast human, animal, and plant diseases [3]. Two main reasons support this. The first is that most of the sequenced genomes belong to pathogens, and the second is that the knowledge of the genome of harmful microbes offers the possibility to identify gene encoding for protein targets, whose inhibition might impair the growth or virulence of the prokaryotic and eukaryotic pathogens [4,5]. Proteins as drug targets are prevalent. Among them, enzymes represent a significant group, since most of them catalyze reactions essential for supporting the central microbe metabolism and, as a consequence, the vitality of the pathogen [6]. The basis of the drug target approach is supported by the following criteria: (a) to identify metabolic pathways which are absent in the host and indispensable for the survival of the pathogen; (b) to recognize enzymes of the metabolic pathway whose inhibition compromise the microbe lifecycle; and, finally, (c) to find compounds which, in vitro (as the first investigation), can interfere with the activity of the identified enzymes [7]. In this context, the genome exploration of pathogenic and non-pathogenic microorganisms has revealed genes encoding for a superfamily of metalloenzymes, known as carbonic anhydrases (CAs, EC 4.2.1.1) [8,9,10,11,12]. CAs catalyze the physiologically crucial reversible reaction of the carbon dioxide (CO_2_) hydration to bicarbonate (HCO_3_^−^) and protons (H^+^) according to the following chemical reaction: CO_2_ + H_2_O ⇋ HCO_3_^−^ + H^+^ [13,14,15]. Many CA inhibitors (CAIs) exist and efficiently inhibit, in vitro, the activity of the CAs encoded by the genome of several pathogens [13,16,17,18]. It has been demonstrated that CAIs are also effective in vivo, impairing the growth and virulence of several pathogens responsible of human diseases, such as *Helicobacter pylori* [19,20,21], *Vibrio cholerae* [22], *Brucella suis* [23,24,25,26], *Salmonella enterica* [27], and *Pseudomonas aeruginosa* [28]. Considering the three major criteria typifying the drug-target approach, it is evident that CAs meet the criteria (b) and (c) entirely. Instead, the criterion (a) is satisfied partly because CAs are ubiquitous metalloenzymes involved in the balance of the equilibrium between dissolved CO_2_ and HCO_3_^−^ in all living organisms. Even if CAs are not species-specific enzymes, they are considered promising drug targets because they offer the possibility to design specific and selective inhibitors for the microbial CAs [13,16,17,18]. For example, the enzyme dihydrofolate reductase (DHFR), although it is ubiquitously expressed in all kingdoms, is a target of several drugs, such as the antibacterial trimethoprim [29]. This enzyme is responsible for the nicotinamide adenine dinucleotide phosphate (NADPH)-dependent reduction of 5,6-dihydrofolate (DHF) to 5,6,7,8-tetrahydrofolate (THF), an essential cofactor used in the biosynthetic pathways of purines, thymidylate, methionine, glycine, pantothenic acid, and *N*-formyl-methionyl tRNA. The bacterial DHFR amino acid sequence has an identity of 30% with the corresponding human protein [29]. Nevertheless, trimethoprim selectively inhibits the bacterial enzyme but not the human DHFR [29].

The CA superfamily is grouped into eight genetically distinct families (or classes), named with the Greek letters α, *β*, γ, δ, ζ, η, θ, and ι [13,14,15,30,31]. In mammalian, for example, 15 CAs are expressed, 12 of which are catalytically active, and all belong to the *α*-class [9,16,32,33,34,35,36,37]. It is interesting to stress that the genome of most pathogens does not encode for a *α*-CA [12,13,14,34,38,39]. This is a unique advantage in finding inhibitors with no inhibitory effect on the CAs from humans and animals. However, when the genome of a pathogen encodes for a *α*-CA, such enzyme (amino acid sequence identity of about 35% respect to the mammalian protein) shows structural differences in the amino acid residues surrounding the catalytic pocket, offering the possibility to tune the CA inhibitors and, hence, a higher probability to inhibit selectively the *α*-CA identified in the pathogen [40,41,42]. Recently, our groups focused on the in vitro inhibition of recombinant *β*-CA (CynT2) from *Escherichia coli* because this CA, localized in the cytoplasm, is indispensable for the growth of the microbe at atmospheric pCO_2_ [43,44]. *E. coli* is a Gram-negative bacterium that, as a commensal microorganism, colonizes the lower intestine of warm-blooded organisms [45,46,47]. In some cases, *E. coli* can act as a severe pathogen able to generate disease outbreaks worldwide [48,49,50], or, as an opportunistic pathogen, which can cause diseases if the host defenses are weakened [51]. Surprisingly, although this enzyme was reported and crystallized two decades ago [43], no inhibition study with any class of CAIs was reported so far. Here, we compare the inhibition profiles of CynT2 with those determined for the *β*-CA from *Vibrio cholerae* and the two human *α*-CA isoforms (hCA I and hCA II), using the sulfonamides and their bioisosteres, which, among the groups of the classical CAIs, generally inhibit the other CAs in the range of nanomolar and have been clinically used for decades as antiglaucoma [29], diuretic [35], antiepileptic [32], anti-obesity [52,53], and anticancer [37] agents.

The goal of the present manuscript is to identify putative compounds, which can eventually go through the other phases of the drug discovery pipeline, such as the structure–activity relationship (SAR), in vitro cell based-tests, in vivo studies, and, finally, the clinical trials, leading to the discovery of new antibacterials.

## 2. Results and Discussion

### 2.1. Production and Enzymatic Activity of the Target CynT2

Generally, the bacterial genome contains genes encoding for at least four CA-classes categorized as α, *β*, γ, and ι [12,14,15,29,38]. The *E. coli* genome analysis allowed the identification of genes encoding for CAs belonging to one of the following classes: *β*, *γ*, and ι. Among them, two *β*-CAs isoforms, indicated with the acronyms CynT and CynT2, were investigated for their physiological function, as well as their involvement in the life cycle of *E. coli*. It has been demonstrated that CynT, catalyzing the CO_2_ hydration, prevents the diffusion of CO_2_ from the cell and produces the bicarbonate essential for the neutralization of the toxic cyanate through the reaction catalyzed by the enzyme cyanase (NCO^−^ + 3H^+^ + HCO_3_^−^ → 2CO_2_ + NH_4_^+^) [54]. Instead, the CynT2 isoform is required for *E. coli* growth at low CO_2_ concentrations (atmospheric pCO_2_), as demonstrated by experiments reported in the literature [43,44]. Thus, with both enzymes being involved in the cellular balance of CO_2_ and bicarbonate, their inhibition was hypothesized to impair bacterial growth and virulence [44].

**Figure 1:**
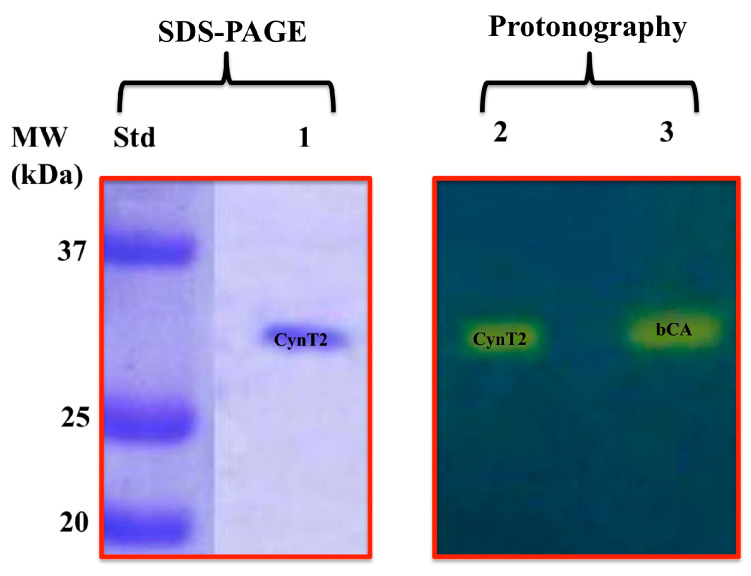
Analysis of the heterologous protein expression on the Coomassie Blue stained gel (SDS–PAGE) and Bromothymol Blue stained gel (Protonography). The purified recombinant CynT2 was eluted from the affinity resin by adding 250 mM imidazole. The yellow band on the protonogram (right of the figure) corresponds to the enzyme activity responsible for the drop of pH from 8.2 to the transition point of the dye in the control buffer. Lane STD, molecular markers (form bottom to the top: 20, 25, and 37 kDa); Lane STD, Molecular markers; Lane 1, purified CynT2; Lane 2, purified CynT2 subjected to protonography; Lane 3, commercial bovine CA (bCA) used as a positive control in the protonography.

Using the recombinant DNA technology, CynT2 was heterologously overexpressed in recombinant form in this work. The biocatalyst was produced as a fusion protein with six tandem histidines (His_6_-Tag) at the N-terminus of the polypeptide chain. The chimeric protein was purified from the soluble cytoplasmic protein fraction of the bacterial host cells, using a nickel-charged resin with high affinity for the polyhistidine sequence. CynT2 showed a high degree of purity, as demonstrated by SDS–PAGE (Figure 1).

After the electrophoretic run, the polyacrylamide gel showed a single protein band (CynT2 as the fusion protein) with an apparent molecular weight of about 29.0 kDa (the theoretical mass of the chimeric CynT2 based on its amino acid sequence was 29.0 kDa) (Figure 1). Following the SDS–PAGE, CynT2 was subject to protonographic analysis, which is a facile new technique developed by us to identify the hydratase activity of CAs on SDS–PAGE [55]. The protonography study demonstrated that the recombinant CynT2 was catalytically active on the polyacrylamide gel, as evidenced by the yellow band due to the hydratase activity in correspondence of the molecular weight of the purified chimeric molecule (Figure 1). The protonogram was obtained by using the recombinant CynT2 from *E. coli*, and the commercial bovine *α*-CA (bCA) was used as a positive control. It is known that the mammalian *α*-CAs are monomeric [9,34]. The protonogram developed from the similar analysis of bCA (mammalian enzyme) showed a single band of activity corresponding to a monomer of about 30 kDa (Figure 1, lane 3). In contrast, all the bacterial *β*-CAs crystallized so far are active as dimers or tetramers [13], with two or four identical active sites. The protonogram of CynT2 showed a band of activity (Figure 1) in the correspondence of its monomer (29.0 kDa). The yellow band appeared in the position of the inactive monomeric form of CynT2, because, at the end of the electrophoretic run, the SDS is removed from the gel. This procedure leads to the rearrangement of the *β*-CA monomers in the gel, and the final result is the reconstitution of the active dimeric or tetrameric forms of the *β*-CA. This also means that CynT2, after the elimination of the SDS from the gel, can refold and generate the active form correctly, as reported for other CA classes present in prokaryotic/eukaryotic organisms [55].

The CO_2_ hydratase activity of the purified and soluble enzyme and its kinetic constants were determined by using the stopped-flow technique. It should be mentioned that, in the initial report of Cronk et al. [43], the enzyme was reported to possess catalytic activity, but the kinetic parameters were not reported, as a rather simple assay was used which does not offer the possibility to easily measure k_cat_ and K_m_ of the enzyme. Thus, we report these measurement and the obtained results are compared with the kinetic parameters of the two mammalian *α*-CA isoforms (h CAI and h CAII) and the *α*-, *β*-, γ-, and ι-CAs from different species of pathogenic bacteria, such *Vibrio cholerae*, *Porphyromonas gingivalis*, *Helicobacter pylori*, and *Burkholderia territorii* (Table 1).

CynT2 resulted in being an excellent catalyst for the CO_2_ hydration reaction (k_cat_ = 5.3 × 10^5^ s^−1^ and a k_cat_/K_M_ = 4.1 × 10^7^ M^−1^ s^−1^). It was inhibited by acetazolamide (**AAZ**), a well-known pharmacological CA inhibitor, with a K_I_ = 227nM (Table 1). Interestingly, all the bacterial enzymes were slightly more active than the human isoform h CAI, which sowed a k_cat_ = 2.0 × 10^5^ s^−1^. From the results reported in Table 1, it emerged that the clinically used sulfonamide had a different sensitivity versus the two human *α*-CAs and the bacterial enzymes. The isoform hCA I was inhibited with a K_I_ = 250 nM, which is very similar to the values obtained for the most bacterial *β*-CAs and *γ*-CAs, whereas the human isoform h CA II, as well as the bacterial *β*-CAs from *H. pylori*, *V. cholerae*, and *B. territorii*, resulted in being more sensitive to the **AAZ** inhibition with a K_I_ in the range from 6.8 to 65 nM (Table 1).

**Figure 2:**
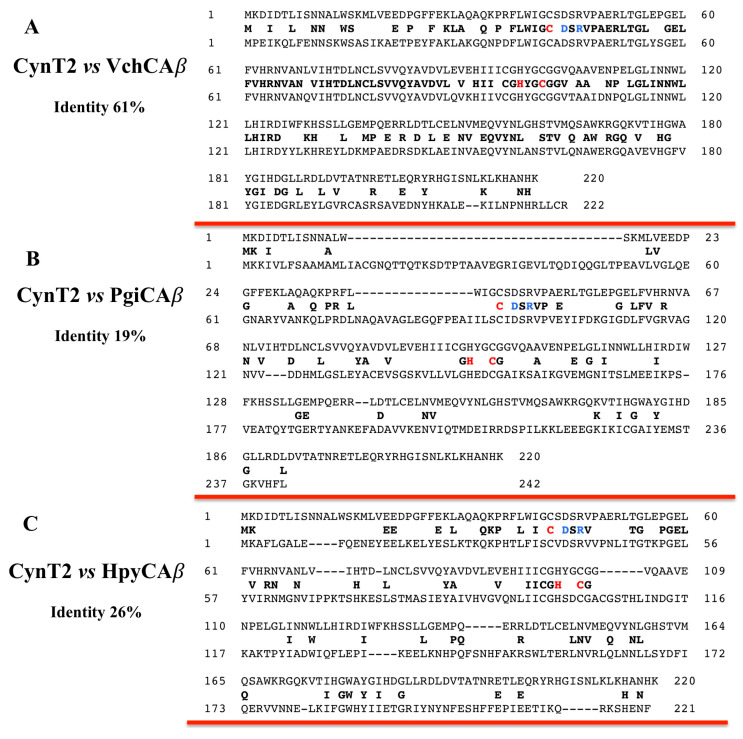
Pairwise comparison of the CynT2 polypeptide chain with VchCA*β* (**A**), PigCA*β* (**B**), and HpyCA*β* (**C**) amino acid sequences, respectively. The identical amino acid residues are indicated between the two aligned amino acid sequences (black bold). The amino acid residues participating in the coordination of the metal ion are indicated in red (Cys, His, and Cys), whereas the catalytic dyad involved in the activation of the water molecule coordinated to zinc (Asp–Arg) is shown in blue. The pairwise alignment was performed with the program Blast Global Align. The accession numbers of the aligned sequences are: EEW0221051.1, CynT2 from *Escherichia coli*; WP_002051193.1, VchCA*β* from *Vibrio cholerae*; WP_012458351.1, PgiCA*β* from *Porphyromonas gingivalis*; WP_000642991.1, HpyCA*β* from *Helicobacter pylori*.

Here, we stress again that the *β*-CAs are metalloenzymes, which use as catalytic metal the Zn(II) ion, which is coordinated by one His and two Cys residues in the enzymatic catalytic pocket (Figure 2). The fourth ligand is a water molecule/hydroxide ion acting as a nucleophile in the enzyme’s catalytic cycle. The comparison of the amino acid sequence CynT2 with those of each *β*-CAs indicated in Table 1 evidenced that, even if these proteins belong to the same class (*β*-class), they are very variegated in the sequence identity, the ratio of the number of identical amino acids in the sequence to the total number of amino acids. Figure 2 shows that CynT2 had 61, 19, and 26% identity compared with the VcCA*β*, PgiCA*β*, and HpyCA*β*, respectively. The catalytic pocket is highly conserved for this CA-class, as also happens for the other CA-classes, but the diversity of the amino acids surrounding the catalytic site influence the biocatalyst kinetic properties and the interaction of the inhibitor, with the enzyme provoking differences in the inhibition constant K_I_ (see Table 1).

### 2.2. CynT2 Sulfonamide Inhibition Profile

**Figure 3:**
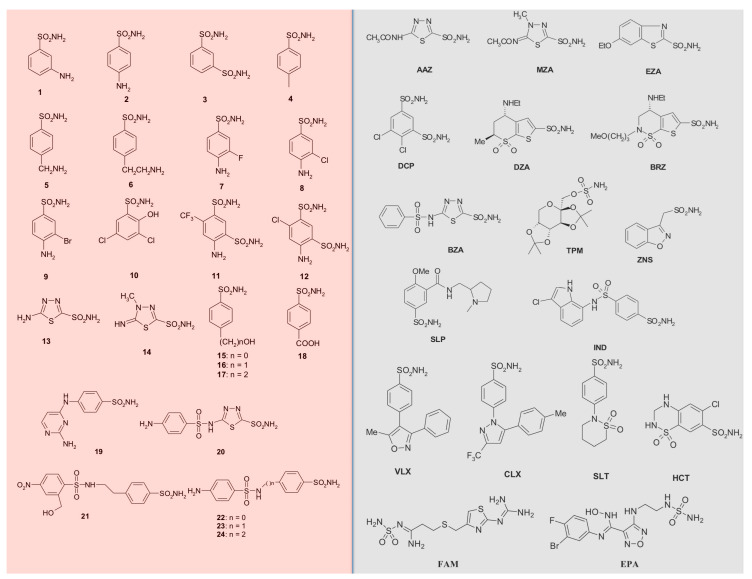
The 42 compounds used to study CynT2 inhibitory behavior. Forty-one sulfonamides and one sulfamate (TPM) were exploited. In red, the series **1**–**24**; in gray, the clinically used drugs.

The exploration of the inhibition profiles of the CAs from different bacterial species is crucial, especially for discovering potent and selective inhibitors, which can be used as a scaffold for designing novel antibacterials interfering with the microbial CA activity and, thus, impairing the microbial life cycle or their virulence without altering the host (human or animal) metabolism. This aspect is fascinating, considering the emergence arisen from the resistance to the existing antimicrobial drugs, which is one of the most severe problems afflicting the human community. Recently, we demonstrated that CynT2 was inhibited efficiently by series of metal-complexing small molecules, including sulfamide, sulfamate, phenylboronic acid, phenylarsonic acid, and diethyldithiocarbamate (K_I_ = 2.5 to 84 µM) (accepted by *Molecules* and in press). Here, a relatively large number of sulfonamides and their bioisosteres have been investigated for their interaction with CynT2. A library of 42 compounds, 41 primary sulfonamides, and one sulfamate, were used as CAIs (Figure 3).

Derivatives **1**–**24** and **AAZ**–**EPA** are either simple aromatic/heterocyclic sulfonamides widely used as building blocks for obtaining new potent and selective families of such pharmacological agents. Table 2 shows a summary of the clinical treatments that use compounds of the series **AAZ**–**EPA** [32,57,58].

Generally, **AAZ**, **MZA**, **EZA**, and **DCP** are systemically acting antiglaucoma CAIs. **DZA** and **BRZ** are antiglaucoma agents that function topically; **BZA** is an orphan drug of this pharmacological class. **ZNS**, **SLT**, and the sulfamic acid ester **TPM** are widely used antiepileptic drugs. **SLP** and **IND** also belong to this class of pharmacological agents, together with the COX2 selective inhibitors **CLX** and **VLX**. **SAC** and the diuretic **HCT** are also known to act as CAIs. In this study, we also considered **FAM** (a competitive histamine H2-receptor antagonist) and **EPA** (an inhibitor of the heme-containing enzyme, indoleamine 2,3-dioxygenase-1 (IDO1)). Most of the sulfonamides, such as the clinically used derivatives **AAZ**, **MZA**, **EZA**, **DCP**, **DZA**, and **BZA**, bind in a tetrahedral geometry to the Zn(II) ion in the deprotonated state, with the nitrogen atom of the sulfonamide moiety coordinated to Zn(II) and an extended network of hydrogen bonds, involving amino acid residues of the enzyme, also participating in the anchoring of the inhibitor molecule to the metal ion [16,18,53]. The aromatic/heterocyclic part of the inhibitor interacts with the hydrophilic and hydrophobic residues of the catalytic cavity [16,53].

Table 3 reports the inhibition profile of CynT2. Moreover, a comparative analysis was carried out, analyzing the CynT2 inhibitory behavior with those obtained for the enzyme VchCA*β* (*β*-CA form *Vibrio cholerae*) [59] and the two human *α*-CA isoforms, hCA I and hCA II [32,60].

From the data of Table 3, the following can be observed:

- The two homologous bacterial enzymes showed a sulfonamide inhibition pattern different from each other. In this context, it is relevant to note that the compounds **22**, **24** and the clinically used inhibitor **SLP** were the best CynT2 inhibitors, showing a K_I_ in the range of 82–97 nM. HTC is the only inhibitor in Table 2 that inhibited the bacterial VchCA*β* with a K_I_ = 87 nM. Exciting is the fact that the inhibitory behaviors of the two bacterial biocatalysts resulted in being highly distinct from those of the two human isoenzymes. The compound **HTC** inhibited the human isoforms, hCA I and h CAII, with a K_I_ value of 290 and 328 nM, respectively. Even if it is challenging to identify selective inhibitors for the bacterial CAs, HTC represents a good example, resulting in medially 3.5 times more efficiently versus the VchCA*β* than the human CAs. However, it is also true that HTC is a harmful inhibitor for the *Escherichia coli* enzyme (K_I_ = 5.0 µM), being 57 times less effective. Other examples are the inhibitors **22**, **24**, and **SLS**, which are 8, 4, and 64 times less effective versus the Vibrio enzyme, respectively, or the inhibitors **13** and **14** with a K_I_ = 68–82 nM are considered potent inhibitors of the Vibrio enzyme. All of these offer the possibility to investigate their molecular interaction with the three-dimensional structures of CynT2 and VchCA*β*, identifying those structural factors responsible for the K_I_ variations. This study allows the design of more efficient and selective inhibitors of the bacterial enzymes that worsen the K_I_ when tested on the two human proteins.
- Among all the compounds investigated, 15 of them showed inhibition constants <1.0 μM for the CynT2. This is the case for compounds **1**, **2**, **3**, **14**, **17**, **18**, **19**, **20**, **21**, **23**, **AAZ**, **MZA**, **EZA**, **DZA**, **BZA**, and **SLT**. These compounds had the K_I_ in the range of 0.2–0.79 μM. Interestingly, some of these CynT2 “strong inhibitors” were mild inhibitors of VchCA*β*, such as **17**, **19**, **21**, **AAZ**, **MZA**, **EZA**, and **DZA** with K_I_ = 2.2–6.2 μM, and, vice versa, compounds **13** and **14** and the **HTC** compound mentioned above resulted in being more sensitive versus the Vibrio enzyme with a K_I_ in the range of 68–87 nM.
- Several compounds of the series **1–24**, such as **4**, **5**, **6**, **9**, **10**, **11**, **12**, **13**, **15**, and **16**, as well as inhibitors of the series **AAZ–EPA**, such as **BRZ**, **TPM**, **IND**, **VLX**, **CLX**, **SAC**, **HTC**, **FAM**, and **EPA,** had a moderate inhibitory effect on the CynT2 enzyme, showing a K_I_ between 1.8 and 8.5 μM. Most of these inhibitors were efficient inhibitors of hCA II (K_I_ = 3–917 nM) and weak inhibitors of the hCA I (K_I_ = 5.8–78.5 μM), except for compound **FAM** (K_I_ = 0.9 µM).
- Some CynT2 inhibitors showed a K_I_ > 10 µM, such as compounds **7**, **8**, and **DCP**, which resulted in a weak inhibitory activity. The weakness inhibitors for the VchCA*β* were **4**, **5**, **6**, **7**, **9**, and **10**. As shown in Table 3, it is apparent that the human *α*-isoenzyme hCA II is efficiently inhibited by all these inhibitors (K_I_ = 38–320 nM) and others with the K_I_ in the range of 3–917 nM. The compound **SAC** represented the only exception having the K_I_ = 5.9 µM. Remarkably, half of the compounds reported in Table 3 resulted in adverse inhibitors for the isoform hCA I. This confirms how important the amino acid surrounding the catalytic pocket is in the inhibition of the enzyme.

## 3. Materials and Methods

### 3.1. Chemicals and Instruments

All the chemicals used in this study were of reagent grade and purchased from Sigma. The Affinity column (His-Trap FF) and the AKTA-Prime purification system were bought from GE Healthcare. The SX20 Stopped-Flow was obtained by the Applied Photophysics. SDS–PAGE and Western blot apparatus were procured from BioRAD.

### 3.2. Heterologous Expression and Purification of the Recombinant Enzyme

The synthetic *Escherichia coli* gene encoding for the CynT2 was synthesized by the Invitrogen GeneArt (ThermoFisher Scientific), a company specialized in gene synthesis, and cloned into the expression vector pET100D-Topo/CynT2. Briefly, the gene was designed to produce the recombinant CynT2 as fusion proteins with a tag containing nucleotides encoding for six histidines (His-Tag) at the amino terminus of neosynthesized recombinant protein. Competent *E. coli* BL21 (DE3) codon plus cells (Agilent) were transformed as described by Del Prete et al. [61]. Isopropyl *β*-D-1-thiogalactopyranoside (IPTG) at the concentration of 1 mM was added to the cellular culture, to overexpress the recombinant CynT2. After growth, the cells were harvested and disrupted by sonication. Cellular extract was purified by using a nickel affinity column (His-Trap FF), which allows the interaction between the matrix functionalized with Ni^2+^ ion and the His-Tag at the N-terminus of the protein. The HisTrap column (1 mL) was equilibrated with 20 mL equilibration buffer (50 mM Tris, 20 mM imidazole, and 150 mM sodium chloride, pH 7.5) at 1 mL/min. The supernatant from the cellular lysate was loaded onto the column, at 1 mL/min, connected with AKTA Prime. The recombinant CynT2 was eluted from the column by fluxing a linear gradient of imidazole (0–300 mM), at a flow of 0.5 mL/min, in a buffer composed of 50mM Tris and 300 mM sodium chloride, pH 7.5. The recovered CynT2 was 90% pure. The protein quantification was carried out by Bradford method (BioRAD) [62]. The CA activity assay was performed as described by Capasso et al. [63]. Briefly, the assay was based on the monitoring of pH variation due to the catalyzed conversion of CO_2_ to bicarbonate. Bromothymol blue was used as the indicator of pH variation. The assay was performed at 0 °C, and a CO_2_-satured solution was used as substrate. The enzyme activity was calculated by measuring the time required for Bromothymol blue to change from blue to yellow. This time is inversely related to the quantity of enzyme present in the sample and allows the calculation of the Wilbur–Anderson units, as described previously [63].

### 3.3. SDS–PAGE and Protonography

A 12% Sodium Dodecyl Sulfate–polyacrylamide gel electrophoresis (SDS–PAGE), prepared as described by Laemmli [64], was used, loading on the gel the recovered CynT2 from the affinity column. The gel was stained with Coomassie Brilliant Blue-R. To perform the protonography, wells of 12% SDS–PAGE gel were loaded with samples mixed with loading buffer not containing 2-mercaptoethanol and not subjected to boiling, in order to avoid protein denaturation. The gel was run at 150 V, until the dye front ran off the gel. Following the electrophoresis, the 12% SDS–PAGE gel was subject to protonography, to detect the yellows bands due to the hydratase activity on the gel, as described previously [55,65,66,67].

### 3.4. Kinetic Parameters and Inhibition Constants

The CO_2_ hydration activity performed by the BteCAι was monitored by using an Applied Photophysics stopped-flow instrument [68]. Phenol red (at a concentration of 0.2 mM) was used as the indicator, working at the absorbance maximum of 557 nm, with 20 mM TRIS (pH 8.3) as buffer, and 20 mM NaClO_4_ (for maintaining constant the ionic strength), following the initial rates of the CA-catalyzed CO_2_ hydration reaction for a period of 10–100 s. To determine the kinetic parameters by Lineweaver–Burk plots and the inhibition constants, a concentration of CO_2_ between 1.7 and 17 mM was used. At least six measurements of the original 5–10% reaction were used to assess the initial velocity for each inhibitor. The uncatalyzed rates were identically determined and detracted from the total observed rates. Stock inhibitor solutions (10–100 mM) were prepared in distilled–deionized water, and dilutions up to 0.01 mM were done with the buffer test. Inhibitor and enzyme solutions were preincubated together for 15 min, at room temperature, prior to assay, in order to allow for the formation of the E-I complex or for the eventual active site mediated hydrolysis of the inhibitor. The inhibition constants were obtained by non-linear least-squares methods, using PRISM 6 and the Cheng–Prusoff equation, as reported earlier [56,69,70], and represent the mean from at least three different determinations. VchaCA*β*, hCA I, and hCA II were recombinant enzymes obtained in-house.

## 4. Conclusions

The *Escherichia coli* *β*-CA (CynT2) was heterologously overexpressed to investigate, for the first time, its inhibition profile with a group of classical CAIs inhibitors. The CAIs considered were the sulfonamides and their bioisosteres and one sulfamate, which generally inhibit the other CAs in the nanomolar range. The recombinant enzyme resulted in an excellent catalyst for the CO_2_ hydration reaction with a k_cat_ = 5.3 × 10^5^ s^−1^ and a k_cat_/K_M_ = 4.1 × 10^7^ M^−1^ s^−1^. The comparison of the inhibition profiles of CynT2 obtained for a bacterial enzyme (VhCa*β*) belonging to the same CA-class of CynT2 and those determined for the two human *α*-CA isoforms evidenced:

1. The compounds **22**, **24**, and the clinically used **SLP** were the best CynT2 inhibitors sowing a K_I_ in the range of 82–97 nM;
2. The inhibition profiles of the four proteins considered (CynT2, VchCA*β*, hCA I, and hCA II) are rather different from each other.
3. All the compounds showing a different behavior versus an enzyme belonging to the *β*- and *α*-class represent good candidates to identify, through the comparison of the three-dimensional structure of the protein with the inhibitor, the structural factors responsible for the K_I_ variations.

The data of the inhibition profile and the structural analysis will help us in the design of antibacterials that can interfere with the CA activity and, thus, with the microbial life cycle or their virulence. This aspect is fascinating, considering the emergence arisen from the resistance to the existing antimicrobial drugs, which is one of the most severe problems afflicting the human community.

## Figures and Tables

**Table 1:** Kinetic parameters for the CO_2_ hydration reaction catalyzed by the human α-CAs: the cytosolic isozymes hCA I and II; bacterial α-CA: VchCAα; bacterial β-CAs: CynT2, VchCAβ, PgiCAβ, HpyCAβ, BsuCA219, BsuCA213, LpCA1 and LpCA2; bacterial γ-CAs: PgiCAγ, VchCAγ; bacterial ι-CA: BteCAι. All the measurements were made at 20 °C, pH 7.5 (α-enzymes), and pH 8.3 (β-, γ-, and ι- enzymes), by a stopped-flow CO_2_ hydrase assay method.

Organism	Acronym	Class	^1^ k_cat_ (s^−1^)	^1^ k_cat_/K_m_ (M^−1^ × s^−1^)	^1^ K_I_ (Acetazolamide) (nM)
*Homo sapiens* ^a^	hCA I	α	2.0 × 10^5^	5.0 × 10^7^	250
	hCA II	α	1.4 × 10^6^	1.5 × 10^8^	12
*Vibrio cholerae* ^a^	VchCAα	α	8.2 × 10^5^	7.0 × 10^7^	6.8
*Escherichia coli*	CynT2	β	5.3 × 10^5^	4.1 × 10^7^	227
*Vibrio cholerae* ^a^	VchCAβ	β	3.3 × 10^5^	4.1 × 10^7^	451
*Porphyromonas gingivalis* ^b^	PgiCAβ	β	2.8 × 10^5^	1.5 × 10^7^	214
*Helicobacter pylori* ^c^	HpyCAβ	β	7.1 × 10^5^	4.8 × 10^7^	40
*Porphyromonas gingivalis* ^b^	PgiCAγ	γ	4.1 × 10^5^	5.4 × 10^7^	324
*Vibrio cholerae* ^a^	VchCAγ	γ	7.3 × 10^5^	6.4 × 10^7^	473
*Burkholderia territorii* ^d^	BteCAι	ι	3.0 × 10^5^	9.7 × 10^7^	65

^1^ Mean from three different assays by a stopped-flow technique (errors were in the range of ±5–10% of the reported values); ^a^ from Reference [56]; ^b^ from Reference [35]; ^c^ from Reference [19]; ^d^ from Reference [15].

**Table 2:** Inhibitor name, commercial name, acronym, and clinical treatment of the CAI clinically used drugs.

Inhibitor Name	Trade Name	Acronym	Clinical Treatment
Acetazolamide	Diamox	**AAZ**	glaucoma, epilepsy, altitude sickness, periodic paralysis, idiopathic intracranial hypertension, diuretic
Methazolamide	Neptazane	**MZA**	glaucoma
Ethoxzolamide	Cadrase	**EZA**	glaucoma, duodenal ulcers, diuretic
Dichlorophenamide	Keveyis	**DCP**	glaucoma, diuretic
Dorzolamide	Trusopt	**DZA**	glaucoma
Brinzolamide	Azopt	**BRZ**	glaucoma
Benzolamide	No generic name	**BZA**	glaucoma
Topiramate	Topamax	**TMP**	epilepsy, migraine
Zonisamide	Zonegran	**ZNS**	epilepsy, Parkinson’s disease, obesity, migraine, bipolar depression
Sulpiride	Dogmatil	**SLP**	psychosis, schizophrenia, anxiety, mild depression
Indisulam	No generic name	**IND**	cancer
Valdecoxib	Bextra	**VLX**	osteoarthritis, rheumatoid arthritis, painful menstruation, menstrual symptoms
Celecoxib	Celebrex	**CLX**	osteoarthritis, acute pain in adults, rheumatoid arthritis, ankylosing spondylitis, painful menstruation, juvenile rheumatoid arthritis
Sulthiame	Ospolot	**SLT**	epilepsy
Saccharin	No generic name	**SAC**	diet
Hydrochlorothiazide	CAPOZIDE	**HCT**	hypertension, congestive heart failure, symptomatic edema, diabetes insipidus, renal tubular acidosis
Famotidine	Pepcid	**FAM**	peptic ulcer, gastroesophageal reflux disease,
Epacadostat	No generic name	**EPA**	cancer

**Table 3:** Inhibition of the human isoforms hCA I and hCA II and the two bacterial *β*-CAs (CynT2 and VchCA*β*) with sulfonamides **1**–**24** and the clinically used drugs **AAZ**–**EPA**.

Inhibitor	K_I_ *(nM)			
	hCA I ^a^	hCA II ^a^	CynT2	VchCA*β* ^a^
**1**	**28,000**	**300**	705	463
**2**	25,000	240	790	447
**3**	79	8	457	785
**4**	78,500	320	3015	>10,000
**5**	25000	170	2840	>10,000
**6**	21,000	160	3321	>10,000
**7**	8300	60	>10,000	>10,000
**8**	9800	110	>10,000	9120
**9**	6500	40	2712	>10,000
**10**	7300	54	8561	>10,000
**11**	5800	63	6246	879
**12**	8400	75	4385	4450
**13**	8600	60	4122	68,1
**14**	9300	19	440	82,3
**15**	5500	80	6445	349
**16**	9500	94	2340	304
**17**	21,000	125	502	3530
**18**	164	46	205	515
**19**	109	33	416	2218
**20**	6	2	726	859
**21**	69	11	473	4430
**22**	164	46	93	757
**23**	109	33	322	817
**24**	95	30	82	361
**AAZ**	250	12	227	4512
**MZA**	50	14	480	6260
**EZA**	25	8	557	6450
**DCP**	1200	38	>10,000	2352
**DZA**	50,000	9	629	4728
**BRZ**	45,000	3	2048	845
**BZA**	15	9	276	846
**TPM**	250	10	3359	874
**ZNS**	56	35	3189	8570
**SLP**	1200	40	97	6245
**IND**	31	15	2392	7700
**VLX**	54,000	43	2752	8200
**CLX**	50,000	21	1894	4165
**SLT**	374	9	285	455
**SAC**	18,540	5959	6693	275
**HCT**	328	290	5010	87
**FAM**	922	58	2769	-
**EPA**	8262	917	2560	-

* Errors in the range of 5–10% of the reported data, from three different assays. ^a^ Human recombinant isozymes and Vibrio enzyme, stopped-flow data from Reference [56]; -, not detected.
